# Correspondence

**Published:** 1987-08

**Authors:** E. C. Hamlyn

**Affiliations:** Rutt House Ivybridge Devon PL21 0DQ


### Correspondence

Sir,

Dr. T. J. David in his article on Bogus Allergy Treatment shows the reader in no uncertain terms that a flourishing private allergy industry has come into existence.

Dr. David makes a scathing attack upon this industry but utterly fails to recognise the beam in our own eye which is the cause of bogus allergy treatment.

Christine Doyle writing in the Daily Telegraph on 10th February 1987, accused the medical profession of being allergic to allergies and by neglect encouraging the growth of bogus allergy clinics.

What is really needed from Dr. David is an answer to some very important questions.

Why is the incidence and morbidity of allergic type illness on a runaway epidemic trend?

What is the relationship between environmental pollution and allergic type illness?

What are the worst forms of pollution in terms of impingement on the health of the individual?

Why is the medical profession only interested in a search for new modes of treating the symptoms of allergy instead of looking to eliminate the cause or causes?

Why is the medical profession refusing to listen to what patients could tell them as a result of successful bogus treatment?

It is axiomatic that in the field of private treatment it is only success which flourishes. Why is bogus treatment of allergy so much more successful than what Dr. David has on offer?

If we can get some of these questions looked at and perhaps answered, then Dr. David's exposé of the current allergy scene in the U.K. will have been worthwhile.

Yours sincerely,

E. C. Hamlyn, MB ChB.

Rutt House

Ivybridge

Devon PL21 0DQ

